# Prognosis Value of Immunoregulatory Molecules in Oral Cancer Microenvironment: An Immunohistochemical Study

**DOI:** 10.3390/biomedicines10030710

**Published:** 2022-03-19

**Authors:** Juan Francisco Peña-Cardelles, José Juan Pozo-Kreilinger, Giovanna Roncador, Jesús Esteban-Hernández, José Ernesto Moro-Rodríguez, Ana Sastre-Perona, Beatriz Castelo-Fernández, José Luis Cebrián-Carretero

**Affiliations:** 1 Department of Basic Health Sciences, Rey Juan Carlos University, 28922 Madrid, Spain; 2 Fellow Oral and Maxillofacial Surgery Department and Prosthodontics Department, School of Dental Medicine, University of Connecticut Health, Farmington, CT 06030, USA; 3 Department of Pathology, Universidad Autónoma de Madrid, Hospital Universitario La Paz, 28046 Madrid, Spain; josejuan.pozo@salud.madrid.org; 4 Head of the Monoclonal Antibody Unit, Spanish National Cancer Research Center (CNIO), 28046 Madrid, Spain; groncador@cnio.es; 5 Public Health and Preventive Medicine Unit, Health Sciences Faculty, Universidad Rey Juan Carlos, 28046 Madrid, Spain; jesus.esteban@urjc.es; 6 Pathological Anatomy Area, Universidad Rey Juan Carlos, 28046 Madrid, Spain; joseernesto.moro@urjc.es; 7 Laboratory of Experimental Therapies and Biomarkers in Cancer, IdiPAZ, 28046 Madrid, Spain; ana.sastre.perona@idipaz.es; 8 Medical Oncology Department, Hospital Universitario La Paz, 28046 Madrid, Spain; beatriz.castelo@salud.madrid.org; 9 Head of Oral and Maxillofacial Surgery Department, Hospital Universitario La Paz, 28046 Madrid, Spain; josel.cebrian@salud.madrid.org

**Keywords:** oral cancer, microenvironment, risk score, PD-1, PD-L1

## Abstract

Objectives: To evaluate the relationship of the immune-checkpoint PD-1/PD-L1 with the clinical evolution of OSCC; to assess survival in OSCC based on the characteristics of TME and histologic risk score; to evaluate the clinical and histopathological relationship of OSCC with immunological TME. Material and Methods: A retrospective study was carried out on 65 samples from patients with OSCC on the floor of the mouth or tongue. Clinicopathological variables and the expression of the biomarkers PD-1, PD-L1, FoxP3, CD4, CD8, CSF1R, and p16 were recorded. The relationship of the clinical and histological variables with the expression of the biomarkers and survival was studied. Results: The univariate and multivariate analysis indicated that positive PD-1 expression was an independent protective factor for survival (overall, disease-free, disease-specific survival) and that high PD-L1 also improved survival. Poorly differentiated histological grades and metastasis were associated with a worse prognosis. Conclusions: PD-1 is a protective survival factor that is maintained independently of PD-L1 expression. High values of PD-L1 expression also improve survival. Higher expression of PD-1 is observed in smaller tumors, and higher expression of PD-L1 is more likely in women. No relationship between the tumor microenvironment and histologic risk score was found to influence the survival patterns studied in the OSCC. There is no evidence of a relationship between the histopathological features and the studied markers, although the positive PD-1 and PD-L1 cases have a lower risk of a high WPOI score, and positive PD-1 expression was associated with a lower DOI.

## 1. Introduction

Oral squamous cell carcinoma (OSCC) is the most frequent neoplasm among head and neck cancers (HNSCC), this group being one of the most frequent groups of cancers in the world [1,2]. The morbidity of its treatment is very high [3], as is mortality, and the survival of patients with this disease at five years is approximately 60% [4,5]. Unfortunately, the average survival of patients with recurrence of the disease or metastasis is approximately 8–10 months [6].

The histopathological characteristics of OSCC were previously studied, and a histologic risk assessment system was developed [7,8], motivated by the high rate of relapses of this neoplasia [9]. Even though there are different immune cells that have an important impact on the tumor microenvironment (TME) and could modify the neoplasia behavior, such as tumor-infiltrating CD8 and CD4 T lymphocytes (TILs), regulatory T cells (Treg), and the recruitment of tumor-associated macrophages (TAMs) [10], the TME that exists around OSCCs is not yet studied in depth [11], improving the understanding of the immune compartment of these tumours is of great interest since clinical trials conducted in recent years evaluated drugs whose therapeutic targets are various immunological checkpoints [12,13,14,15,16,17].

The latest drug approved by the Food and Drug Administration (FDA) as an immunotherapeutic against HNSCC is pembrolizumab. Candidates for HNSCC immunotherapy are often studied using the combined positive score (CPS) to evaluate the expression of PD-L1 [13,14]. This system is used in other neoplasias (i.e., urogenital carcinoma, cervical cancer, gastric adenocarcinoma), but for OSCC, the tumor proportion score (TPS) is used. Its current indication is the measurement of PD-L1 in non-small cell lung carcinoma (NSCLC) and other types of cancer. Currently, due to the importance of those patients who have a positive response to pembrolizumab when CPS is ≥1 in HNSCC, as in the case of OSCC, the use of CPS and the biomarker of the 22C3 clone is recommended by the FDA as a complementary diagnostic method for HNSCC [13,18].

The present study of the expression of the immunoregulatory molecules PD-1, PD-L1, FoxP3, CD4, CD8, CFS1R, and p16 in the TME is the first study on the immune checkpoint PD-1/PD-L1 that records the CPS, TPS, and expression intensity and that also studies the relationships between these expression measures and histologic risk. The objective of this study is to evaluate the relationship of the immune-checkpoint PD-1/PD-L1 with the clinical evolution of OSCC; to assess survival in OSCC based on the characteristics of TME and histologic risk score; and to evaluate the clinical and histopathological relationship of OSCC with immunological TME.

## 2. Materials and Methods

This was a retrospective epidemiological study that included 65 patients diagnosed with OSCC on the anterior tongue or floor of the mouth, treated at the Oral and Maxillofacial Surgery Department and the Pathological Anatomy Department of La Paz University Hospital (HULP) in Madrid between 2010 and 2015.

### 2.1. Collection of Clinical Data

Inclusion criteria: Patients with an anatomopathological diagnosis of primary OSCC after surgical resection on the anterior tongue (C02.0, C02.01) and/or floor of the mouth (C04) between 2010 and 2015.

Exclusion criteria: (1) Patients treated with oncological therapy, either pharmacological or radiotherapeutic, before surgical removal of the tumor. (2) Patients with a positive diagnosis of human immunodeficiency virus. (3) Patients whose diagnosis was an oral carcinoma with microinvasion. (4) Patients with missing relevant information or with insufficient histological material to be able to perform the histopathological analysis.

The clinical variables sex, age, smoking habit (never, current, former), drinking habit (never, current, former), the primary location of the tumor, the dates of diagnosis and treatment (surgery, radiotherapy, chemotherapy), the presence of recurrences (local, regional, distant), and the presence of oral potentially malignant disorders (OPMD) were collected based on the latest classification of these lesions [19].

The characteristics of the neoplasia, such as tumor size and the presence of regional or distant metastases, were recorded according to the latest tumor–node–metastasis (TNM) classification of the head and neck region of the American Joint Committee on Cancer (AJCC) [20].

### 2.2. Preliminary Anatomopathological Analysis and Selection of Histological Blocks

The tissue samples came from the Pathology Department of the HULP. Each sample was analyzed through a multiviewer light microscope at the same time by three independent observers (JFPC, JJPK, JEMR). Prior to the evaluation of the samples, the three observers came to a consensus on how to determine the histological features of each sample and on a comparison for immunohistochemical interpretation. They studied the samples in random order and while blinded to the clinical information of the patient.

Based on the histopathological characteristics, the most representative paraffin blocks of each case were selected. We previously assessed all the histological samples, confirming that they were an objective and representative sample of the tumors.

Histological features were evaluated using the histologic risk assessment model [7,8]. Following the definitions laid out for this histologic risk model, the lymphocytic host response (LHR), the worst pattern of invasion (WPOI), and PNI were considered. After categorization according to this classification, the tissues were divided into three groups (risk 0–1, risk 2–3, risk 4–7) to facilitate statistical analysis.

The histological grade of the tumor was also recorded, classifying it as poorly (PD), moderately (MD), or well-differentiated (WD) according to the criteria of the World Health Organization (WHO), as well as whether the tumor presented vascular and/or lymphatic invasion [21].

To record the depth of invasion (DOI), we first determined whether the lesion was exophytic or ulcerated. Then, a horizontal line was drawn delimiting the basal membrane and a vertical line (“plumb line”) from this to the invasion front of the tumor, to classify it as a mildly (≤5 mm), moderately (>5 mm and ≤10 mm), or deeply invasive lesion (>10 mm) [22].

### 2.3. Immunohistochemistry

Immunohistochemical staining was performed as follows: two-meter-thick sections were prepared from formalin-fixed, paraffin-embedded tissue blocks. After this, all sections were oven-dried overnight at 60 °C. The sections were placed in a Bond Max Automated machine.

Immunohistochemistry Vision Biosystem (Leica Microsystems GmbH, Wetzlar, Germany) according to the following procedure. First, the tissues were deparaffinized and pre-treated with Epitope Retrieval Solution 2 (EDTA buffer pH 8.8) at 100 °C for 20 min. After washing, for a period of 10 min, a peroxidase blocking was carried out with the Bond DC9800 polymer detection kit (Leica Microsystems GmbH, Wetzlar, Germany).

The tissues were washed again and then incubated with the primary antibodies for 30 min. Tissues were incubated with the polymer for 15 min and then with DAB-Chromogen for 10 min. In parallel, positive and negative controls of human tonsils were performed. As controls for the technique, incubations omitting the specific antibody or containing unrelated antibodies were used.

Primary antibodies were used following this technique: PD-1 (antibody type: mouse monoclonal; clone name: NAT105, dilution: 1:4 supernatant; source: CNIO), FoxP3 (antibody type: mouse monoclonal; clone name: 236A, dilution: prediluted; source: CNIO), CD4 (antibody type: mouse monoclonal; clone name: 4B12, dilution: prediluted; source: DAKO), CD8 (antibody type: rat monoclonal; clone name: NOR132H, dilution: 1:5 supernatant; source: CNIO), CSF1R (antibody type: mouse monoclonal; clone name: FER216, dilution: 1:20 supernatant; source: CNIO).

The immunohistochemistry for PD-L1 (antibody type: mouse monoclonal; clone name: 22C3, prediluted, source: DAKO (Agilent Technologies, Santa Clara, CA, USA)) was performed on 3-µm tissue sections that had been deparaffinized in an oven at 60 °C for 20 min and unmasked in buffer of low pH at 97 °C for 20 min, all of which took place in the Autostainer Link 48 system with EnVision FLEX reagents (K8002) (DAKO, Agilent Technologies, Santa Clara, CA, USA). The tissue was incubated with primary antibody for 30 min, endogenous peroxidase inhibitor for 10 min, secondary antibody for 30 min, diaminobenzidine for 10 min, and hematoxylin for 7 min, followed by buffer, distilled water, an ascending series of alcohol, and xylol.

For the p16 biomarker (antibody type: mouse monoclonal; clone name: E6H4; source: Roche (Roche diagnostics, Basel, Switzerland)), histological sections were made at 3 µm and then deparaffinized in an oven at 70 °C for 15 min, followed by histological unmasking in pH buffer high at 95 °C for 20 min. The section was incubated in the primary antibody for 30 min, followed by washing in buffer. Then, endogenous peroxidase was inhibited for 10 min, and the secondary antibody was added for 30 min. After this came another buffer wash, diaminobenzidine for 10 min, hematoxylin for 7 min, buffer, distilled water, ascending alcohol, and xylol. Staining was carried out using an automated OMNIS system (DAKO, Agilent Technologies, CA, USA).

The biomarkers PD-L1 and p16 were also detected with positive and negative controls in the study samples.

### 2.4. Immunohistochemical Observation

Immunohistochemical interpretation was performed by the three observers with the same initial criteria. The area studied was the peripheral intratumoral component. Nuclear staining for FoxP3 was considered to identify regulatory T cells. CSF1R in the membrane and cytoplasm was considered to identify tumor-associated macrophages (TAMs). CD4 and CD8 at the membrane level were considered to study tumor-infiltrating lymphocytes (TILs). PD-1 and PD-L1 were considered expressed when the staining was only at the membrane level.

For the objective scoring of the expression of the biomarkers, the samples were observed under a light microscope at magnifications of 10×, 20×, and 40× for discernment in cases of weak staining. We calculated the percentage of expression of the biomarkers except for PD-L1, for which the following three scoring systems were used:

1. TPS: defined as the percentage of viable tumor cells showing partial or complete membrane staining for PD-L1 relative to all viable tumour cells present in the sample (positive and negative). Cytoplasmic staining, as well as infiltrating immune cells, normal cells, necrotic cells, and debris, do not go into the score.

2. CPS: defined as the number of PD-L1-staining cells (tumor cells, lymphocytes, macrophages) divided by the total number of viable tumor cells multiplied by 100. Although the result of this calculation can exceed 100, the maximum score is defined as 100. CPS was stratified into three groups: <1, ≥1, and ≥20.

3. Intensity: recorded with the classic classifications of negative, weak, moderate, and strong.

In the case of PD-1, tumors with an expression percentage > 0% were considered positive. Tumors with CPS > 1 and TPS ≥ 5% were considered positive for PD-L1.

To assess the positivity of human papillomavirus (HPV) through p16, the criteria of the College of American Pathologists were used. Positive cases were considered those whose tumor tissue showed nuclear and cytoplasmic immunoreactivity in ≥70% of the cells [23]. After the immunohistochemical study, positive cases were re-evaluated by PCR. p16^+^ cases by both immunohistochemistry and PCR were considered positive.

The other biomarkers were categorized for statistical analysis as follows: CD8 (expression of 0–10% (mild), 10–50% (moderate), ≥50% (severe)); CD4 in three groups (tertiles): 5–25%, 25–35% and 35–50%; FoxP3 in four groups: 1–5%, 5–10%, 10–20%, and 20–100%; and CSF1R according to the cutoff median in two groups: 0–8% and 8–100%.

### 2.5. Survival

Patient survival data were collected from clinical records. The follow-up period was from the date of diagnosis to the closest month to the results. Outcomes were defined as follows: death from oral cancer, death from other causes, recurrence (local, regional, distant), and alive without recurrence. According to these results, we considered three survival definitions: disease-specific survival (DSS), where only death from oral cancer was considered an event; disease-free survival (DFS), where either recurrence (any type) or death from oral cancer (but not death from another cause) was considered an event; and overall survival (OS), where events were defined as death by any reason.

### 2.6. Statistical Analysis

Normally distributed continuous variables were described as a mean and standard deviation; non-normally distributed continuous variables were described using the median and interquartile interval. Discrete variables are presented as count and percentage. Differences in continuous variables were tested using Student’s t-test if normality assumption was met or the Wilcoxon Rank Sum if not. Homogeneity between proportions was tested using Pearson’s chi-squared or Fisher’s exact test, as appropriate.

Kaplan–Meier survival functions were plotted using the same cutoff and were compared using the Wilcoxon–Breslow–Gehan test. Incidence rates for the three different survival definitions (DSS, DFS, OS) were estimated as the number of cases divided by the number of person years of follow-up. The associations between demographic and clinical factors and events for each event definition were analyzed using univariate and multivariate Cox proportional hazards models. Multivariate models included those variables associated with a new event in univariate analysis (*p* < 0.15) and those considered relevant regardless of the *p*-value. Proportional hazard assumption was tested using the Grambsch–Terneau test. All models fulfilled the proportionality assumption.

Bivariate correlation between biomarkers was estimated using non-parametric Spearman’s rank-order correlation, with *p*-values adjusted for multiple testing with Holm´s method.

Survival analysis was performed using the R survival package (version 3.2-13). Kaplan–Meier curves were plotted using the ggsurvplot function from the survminer package (version: 0.4.8). Incidence rates and 95% confidence intervals were estimated using the SurvRate function from the biostat3 package (version 0.1.5.9). All analyses were performed in R (version 4.0.3, GNU GPL-3, R Foundation for Statistical Computing, Vienna, Austria.) via RStudio (version 1.3.959, GNU GPL-3, RStudio: Integrated Development Environment for R. RStudio, PBC, Boston, MA URL).

## 3. Results

### 3.1. Sample Selection

The initial sample consisted of 84 patients with a primary diagnosis of OSCC in the floor of the mouth and/or mobile tongue after consulting the database of the Oral and Maxillofacial Surgery Service between 2010 and 2015. In the final selection, 19 patients were excluded (three with non-OSCC neoplasms, three without enough histological material for the study of histopathological features, six with oral carcinoma with microinvasion, and seven with OSCC not in the floor of the mouth or anterior tongue region), leaving a total of 65 to be studied (Supplementary Figure S1).

### 3.2. Clinical and Histopathological Characteristics

The clinical and histopathological characteristics of the patient sample are reflected in Table 1. The study finally included 40 men (62%) and 25 women (38%) with a mean of 65 years. The mean age was 6 years higher in women (95% CI −2.6; 10.6, *p*-value = 0.23). Forty-eight percent had never smoked, and this proportion was higher in women than in men (76% vs. 29%, *p* = 0.001). The proportion of nondrinking women was also higher (96% vs. 50%, *p* < 0.001). The most frequent primary location was the tongue (65%), followed by the floor of the mouth (26%). In 9.2% of cases, the tumor was present in both, so 74% of patients had a tumor on the tongue and 35% on the floor of the mouth. Sixty-six percent of tumors were stage III or IV, with a similar distribution in both sexes. A total of 15% of the patients (*n* = 10) presented OPMD before the diagnosis of OSCC, with five cases of lichen planus, three cases of leukoplakia, two cases of hyperplastic candidiasis, and one case of chronic actinic cheilitis.

From a histopathological point of view, 91% of the tumors were well or moderately differentiated, without differences by sex, and the evaluation of the pattern of WPOI showed that 89% of these were in types 1 to 4. Perineural invasion was present in 40% of patients (28% women, 48% men, *p* = 0.118), and vascular invasion was present in 5 (7.7%). Lymphocytic infiltration was complete in five cases (7.7%), moderate or intermediate in 25 (38%), low in 33 cases (51%), and null in 2 (3.1%).

The median DOI was 9 mm. A total of 35% of the patients had mild (≤5 mm), 29% moderate (6–10 mm), and 37% deep (≥10 mm). Four cases had affected margins. The distribution was not different according to sex.

Regarding the risk score, larger tumors (T3 + T4) and a drinking habit were associated with higher risk scores (6 and 7).

### 3.3. Biomarkers

The percentage of expression of each biomarker in the whole sample and each sex is shown in (Supplementary Table S1). The distribution was only normal for CD8, so the correlations were analyzed using the Spearman rho coefficient (Supplementary Table S2). Although none of the correlations was significant in our analysis, the correlation between FoxP3 and CD4 (rho = 0.34, *p* = 0.114) was noteworthy. PD-L1 was also positively correlated with PD-1 (rho = 0.25, *p* = 0.737) and with CSF1R (rho = 0.28, *p* = 0.452), but the correlations were not significant after *p*-value correction for multiple testing.

### 3.4. PD-1 and PD-L1

The median expression of PD-1 was 1%, and 80% of the tumors we analyzed (75% in men, 88% in women, *p* = 0.34) were positive (>0%).

PD-L1 expression was evaluated using three scoring systems: CPS, TPS, and staining intensity. The first two had higher medians among women (CPS: 1 vs. 3, *p* = 0.138; TPS: 0% vs. 1%, *p* = 0.037). After categorization according to the previously suggested cutoffs for each scoring system (CPS > 1, TPS ≥ 5%), CPS was classified as positive to the 54%, while only 34% of the samples were over >5% for TPS, with 23% of discordant pairs (CPS > 1 and TPS < 5%, Mcnemar´s test, *p*-value 0.0037). Proportion of subjects above the cutoff was slightly higher in women in both cases (CPS > 1: 64% vs. 48%, *p* = 0.194; TPS ≥ 5%: 48% vs. 25%, *p* = 0.057). The evaluation of staining intensity using the classical criterion (four categories) revealed a slightly higher proportion of women in the moderate and strong categories (*p* = 0.25). See Supplementary Table S1.

### 3.5. PD-1 Expression

PD-1 positivity was associated with Nil/Low-type lymphocytic infiltration compared to PD-1-negative cases (46% vs. 85%, *p* = 0.0295), with a lower DOI (median DOI 8 vs. 12, *p* = 0.017) (Supplementary Table S3). Therefore, PD-1-positive tumors were more often in the less invasive category (39% vs. 17%, *p* = 0.087) and more often were small (67% vs. 33% in category T1 or T2, *p* = 0.073).

### 3.6. PD-L1 Expression

High PD-L1 expression values (TPS ≥ 5%) were associated with a greater median depth of invasion (10 mm vs. 7 mm, *p* = 0.17) but with a more favorable WPOI (WPOI-5: 0% vs. 16%, *p* = 0.085). When the criterion used was CPS > 1, a similar relationship was observed in the WPOI (2.9% vs. 20%, *p* = 0.043). CPS > 1 was also correlated with a lower histological risk score (score 4–7: 47% vs. 26%, *p* = 0.043) and a higher proportion of local recurrences (40% vs. 17%, *p* = 0.039) (Supplementary Table S3).

### 3.7. Other Biomarkers

**Figure 1:**
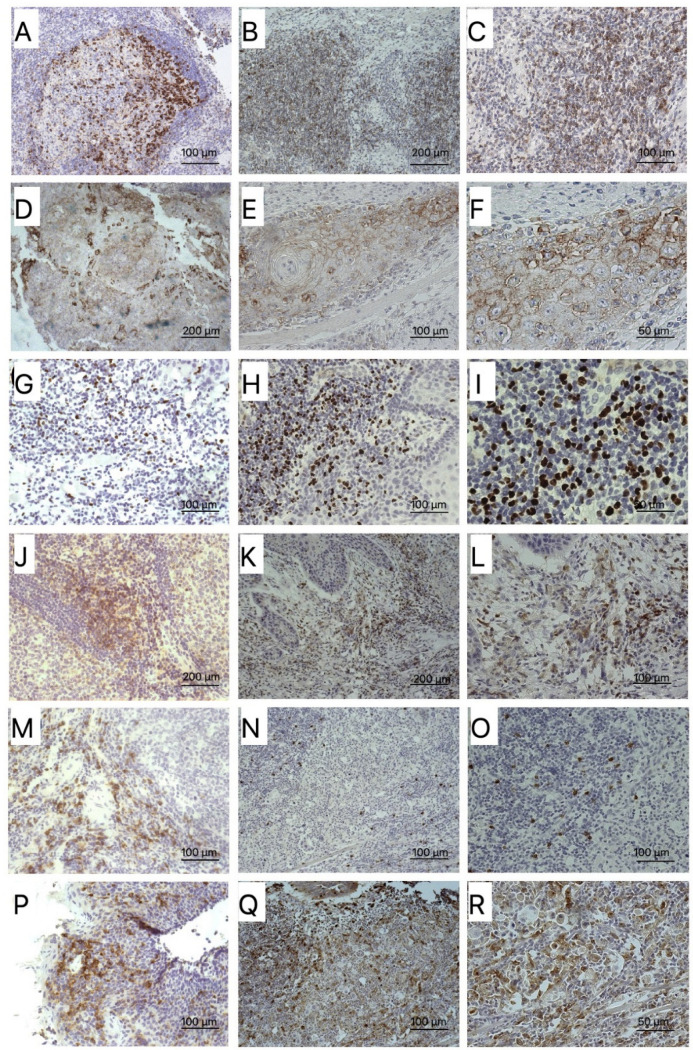
(**A**) Positive control of PD-1 with 10× magnification. (**B**,**C**) Histological section of the expression of 7% PD-1 with 10× and 20× magnification. (**D**) Positive control of PD-L1 with 10× magnification. (**E**,**F**) Microphotograph showing 75% (TPS) PD-L1 expression (CPS: 85) at 20× and 40× magnification. (**G**) Positive control of FoxP3 with 10× magnification. (**H**,**I**) Image by light microscopy (20× and 40×) of histological sections of a sample whose total expression of FoxP3 was 6%. (**J**) Positive control of CD4 with 10× magnification. (**K**,**L**) 10× and 20× images of the CD4 biomarker in a sample with a total expression of 35%. (**M**) Positive control of CD8 with 10× magnification. (**N**,**O**) Histological section of the expression of 5% CD8 with 10× and 20× magnification. (**P**) Positive control of CSF1R with 10× magnification. (**Q**,**R**) Microphotograph showing 20% CSF1R expression at 10× and 20× magnification.

Supplementary Table S1 show the descriptive statistics for each biomarker according to sex. We found a higher expression in FoxP3 among women (median 15% vs. 10%, *p* = 0.073). Fifty-five percent of the sample expressed FoxP3 in more than 10% of the tumor cells (45% men, 72% women, *p* = 0.061). p16 was found in three samples, but only two were positive for HPV on PCR, while CSF1R was found in 42% of the samples, with a similar distribution in both sexes. CD4 expression was slightly higher among women (median 30% vs. 20%, *p* = 0.223), and 32% of the analyzed samples showed expression higher than 35% (25% men, 44% women, *p* = 0.126). CD8 was expressed similarly in men and women (median 30%). Figure 1 show representative histological sections of the different markers in OSCC tissue and their respective control tissues.

### 3.8. Survival Analysis

During a median follow-up (OS) of 73 months (p25–p75: 45–96), 32 deaths (49%) by any cause (42% by OSCC) were observed. None were lost to follow-up. The incidence rate was 8.4 events per 100 person years (95% CI: 5.7, 11.8) for the whole group, and it was significantly higher among men (11.2 [7.2,16.7] events/100 persons years compared to a woman (4.7 [2.03–9.29] events/100 persons years), *p* = 0.032).

**Figure 2:**
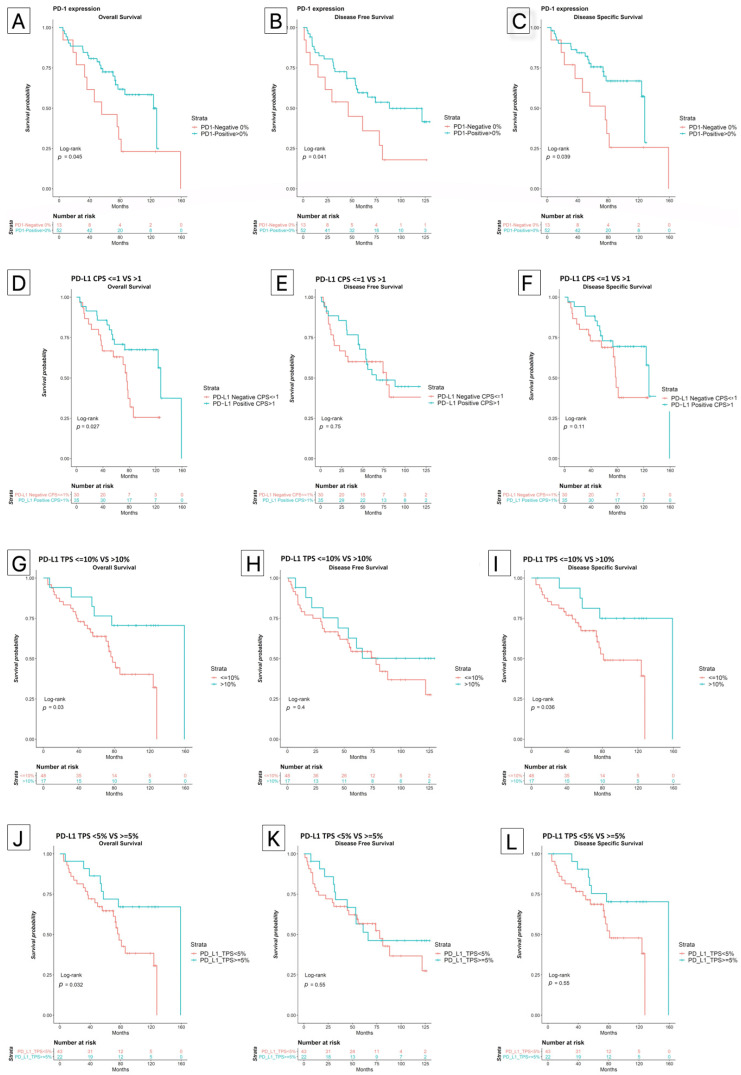
Kaplan–Meier curves. (**A**–**C**) show the survival curves of the PD-1 negative and positive patients. A greater survival is observed in positive PD-1 cases. The remaining graphs show the expression of PD-L1. (**D**–**F**) reflect categorization by CPS ≤ 1 vs. >1; (**G**–**I**) reflect categorization by TPS < 10% vs. >10%; while (**J**–**L**), TPS < 5% vs. >5%. It is clear that longer OSS and DSS are observed when the PD-L1 expression values are higher.

Kaplan–Meier analysis showed an increased OS hazard for those who were PD-1 negative and for those who were PD-L1 negative whether TPS ≥ 5% or CPS > 1 was used (Figure 2). TPS-negative but not CPS-negative patients also had a higher cumulative hazard for DSS.

Univariate Cox models (Supplementary Table S4) showed that smoking, metastasis, a poorly differentiated tumor, and WPOI score 4–5 were associated with hazard of death (OS and DSS models), while women had a lower hazard rate (HR 0.356 [0.14,0.89], *p* = 0.028) compared to men.

Among biomarkers (Table 2), PD-1 positivity was associated with a protective effect (DSS model, HR 0.43 [0.19,0.98], *p* = 0.044; OS model, HR 0.47 [0.22,1.02], *p* = 0.05; DFS model, HR 0.47 [0.22–0.99], *p* = 0.047). PD-L1 positivity (TPS ≥ 5%) was also associated with a better prognosis (DSS model, HR 0.42 [0.17,1.05], *p* = 0.063; OS model, HR 0.41 [0.17,0.95], *p* = 0.038). The results were similar when positivity based on CPS (>1%) was used instead (DSS model, HR 0.53 [0.24,1.17], *p* = 0.12; OS model, HR 0.44 [0.213,0.927], *p* = 0.031).

On the other hand, only the presence of metastasis and a poorly differentiated tumor was associated with a higher risk of recurrence (any type) or death from any cause (DFS model). In this model, only PD-1 positivity showed a protective role (HR:0.47 [0.23,0.99], *p* = 0.047).

**Figure 3:**
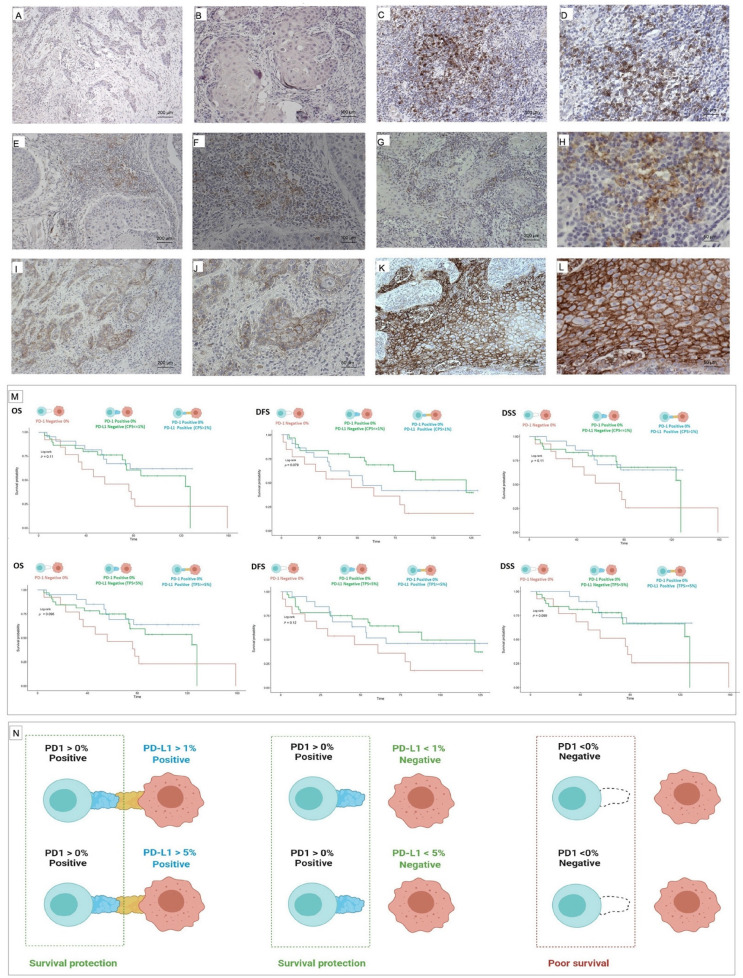
(**A**,**B**) A 5× and 10× histological section of a moderately differentiated tongue OSCC sample. Its expression of the PD-1 biomarker was null, making it negative for PD-1 (<0%). (**C**,**D**) A 10× and 20× histological section of a moderately differentiated tongue OSCC sample was observed, with scarce lymphocytic infiltration and a PD-1 expression of 15%, therefore being positive (>0%). (**E**,**F**) A case in the tongue is shown with a magnification of 10× and 20×, moderately differentiated and with intense lymphocytic infiltration and a PD-L1 expression < 1%. (**G**,**H**) Moderately differentiated case with intense lymphocytic infiltration whose PD-L1 expression was >1%. 10× and 40× magnification. (**I**,**J**) Case of PD-L1 < 5% at 10× and 20×. The expression, in this case, was 2%. (**K**,**L**) In this sample, PD-L1 > 5% is observed at 10× and 20×. The expression of PD-L1, in this case, was 80%, and a granular linear membrane staining pattern was exhibited. (**M**) Kaplan–Meier curves showing that PD-1-positive cases (>0%) and PD-L1 cases with greater and lesser positivity cuts of 1% and 5% show a better prognosis than cases that are PD-1 negative (<0%). (**N**) Illustration showing the protective role of survival by the positive presence of PD-1 expressed in the lymphocyte membrane. Said survival protection remains independent of the values of expression and the degree of positivity of PD-L1 expressed by the tumor cell. The worst prognosis for survival is seen when PD-1 expression is negative.

Stratified analysis showed that the protective role of PD-1 positivity was not modified by the PD-L1 status in any model, regardless of the cutoff definition (TPS > 5% or CPS > 1). See Figure 3.

Regarding biomarkers, only p16 was positivity associated with a poor prognosis in the univariate OS model (HR 4.45 [1.038,19.1], *p* = 0.044), but only two patients were HPV positive.

### 3.9. Multivariate Analysis

After multivariate analysis (Table 3), the presence of metastasis and a moderate or poorly differentiated tumor was associated with poor prognosis in all the survival models. A worse type of WPOI also increased the hazards for all the outcomes, while PD-1 positivity was a protective factor, even after the adjustment by sex and PD-L1, and especially in the DFS model (HR 0.36 [0.14,0.93], *p* = 0.034).

## 4. Discussion

Our research indicated that the expression of PD-1 in the TME has an independent protective role in all survival models evaluated. PD-L1 positivity also exhibited protective effects on OS and DSS by univariate analysis. PD-L1 expression values above 10%, were associated with protective effect on DSS (*p* = 0.047) and OS (*p* = 0.037). The significance of the PD-L1 protective effect was increased for CPS values between 20 and 100 on both DSS (*p* = 0.024) and OS (*p* = 0.019).

In support of our data, Kogashiwa et al. also found a significant association between PD-L1 positive tumors (those expressing greater than 5%) and greater DFS and OS [24]. Additionally, Hanna et al. studied a population of young patients with OSCC and found that PD-L1 expression ≥ 10% was associated with greater survival and a lower risk of recurrence in women [25]. Moreover, Ahn et al. found that higher expression of PD-L1 was associated with a favorable OS prognostic factor [26].

A higher expression of PD-L1 in females and a lower rate of recurrence in females with higher PD-L1 was found in other studies [24,25,27,28]. Our data was in agreement since we found a higher median expression of PD-L1 in women. This result was also confirmed in a recent meta-analysis [29]. Concordantly, we also found that female sex was a protective factor (DSS *p* = 0.028, OS *p* = 0.015) in univariate analysis. However, the strength of this association weakened (more than 30% change in the coefficients) with the entry of PD-L1 TPS > 10% into the model. Nevertheless, the protection associated with female sex did not weaken with PD-1 positivity, smoking, or drinking (all of them more frequent in women). The data supports an independent protective role for the expression of PD-L1 in this group.

Despite the above, a relevant finding is that PD-1 was a protective factor for survival regardless of the value of PD-L1, giving it a determining role and an effect that is maintained after multivariate adjustment (HR: 0.36 [0.14,0.93], *p* = 0.034 (Table 2 and Table 3, Figure 3). These results are similar to those obtained by Kikuchi et al. They found an association of PD-1 in tumor-infiltrating cells with a better prognosis (HR: 0.2, *p* = 0.02) [30]. In our study, the patients who were PD-1 negative and PD-L1 negative also showed a worse survival prognosis by Kaplan–Meier analysis, which seems to support the protective role of PD-1 positivity (Figure 3). The protective effect of PD-1 is mainly observed in OS and DSS. However, in DFS, the protective effect is decreased perhaps due to the fact that tumor recurrence is considered an event in DFS, and positive expression of PD-1 and PD-L1 has been related to increased local recurrences by some authors [31].

The prognostic roles of PD-1 and PD-L1 in OSCC are controversial. Heterogeneity in the results from different studies may be due to the use of different biomarkers to study the PD-1/PD-L1 immune checkpoint and the positivity cutoffs for these biomarkers. Cut-off points at 5% are the most frequently used for PD-L1 [24,25,29,31,32,33,34,35,36], although some authors use 1% [26,29,36,37,38] and, with less frequently, 10% [37,39,40]. The choice of cutoff is mainly based on statistical criteria or the cutoff points of previous studies.

Cutoff points for PD-1 analysis are more variable, with some authors that estimate PD-1 positivity when it is >30% in the sample [30] while others estimate it when it is >1% [36]. We estimated PD-1 positivity when it was >0%, and although an overestimation can be assumed, our mean PD-1 expression percentages (80%) are not very different from other studies such as Mauruse et al., which was 61.1%, or Kouketsu et al., which was 68.9%.

The present investigation advocates the division of the PD-L1 positivity study into three groups based on the CPS classification used in clinical trials and taking into account the expression of >1 CPS, as this is associated in the long term with better prognosis with the use of immunotherapy [41].

The clinical advantages of the use of pembrolizumab demonstrated by different clinical trials and the establishment of CPS as a measure to record the expression of PD-L1 with a clinical approach [13,14] prompted us to analyze the clinical therapeutic possibilities by recording the CPS and the TPS for comparison. In addition, we also recorded the staining intensity, which is assessed less often. This should allow for a better comparison of our results with those of other studies.

In addition, we used the monoclonal antibody PD-L1 22C3 to study the expression of PD-L1, as it is used in pembrolizumab assays in OSCC. Few studies recorded the expression of this biomarker, such as [29,37], but both studies recorded only TPS and not CPS. One study, [40], using both TPS and CPS, found no relationship between PD-L1 and survival. However, the PD-L1 expression pattern showed a relationship with survival, despite the use of a biomarker different from the 22C3.

As indicated above, 22C3 is the biomarker used in clinical trials to evaluate pembrolizumab response in cancers. Alternative biomarkers are used to study if PD-L1 is expressed, for example, E1LN3. Differences in PD-L1 detection between 22C3 and E1LN3 were reported by De Vicente et al. In a clinical trial, which compared the use of PD-L1 22C3 (Dako), 28-8 (Dako), Ventana SP163, and Ventana SP142, the staining was similar for all biomarkers except for SP142 which was lower [42]. However, in another study, 22C3 was less sensitive in tumor and immunological cells. These results suggest that assay results are not interchangeable and clearly indicate the need to systematize the assays [43].

The intensity of expression of the PD-L1 biomarker was also recorded in this study. A higher intensity of expression is mainly associated with worse survival and the presence of regional metastases [44,45]. In our univariate analysis, moderate intensity of PD-L1 expression was associated with better survival (*p* = 0.063) than no expression.

To understand this discrepancy, we studied the whole histological block since the expression of biomarkers of the TME, such as PD-L1, presents great variability within the same tumor [46,47]. In fact, a previous finding that we verified is the most evident expression of PD-L1 on the tumor invasion front [46]. Many studies with results different from ours are based on the study of samples using tissue microarrays (TMAs) [34,35,38,44,48,49,50], which have great limitations. These biomarkers have heterogeneous expression in different regions of the tumor tissue and often leave out, for example, the invasion front. TMAs are inadequate to reproduce the clinicopathological correlations that exist in an analysis of complete sections [46,47]. Thus, studying the entire sample to correctly record the tumor area and its microenvironment [51] did bring some advantages.

In addition, differences in methodology may account for different results. Variations in tissue fixation times, the thickness of histological sections, application of biomarkers, timing from sample collection, and other features affect the sensitivity and specificity of the antibodies used [52]. One study concluded that cell membrane biomarkers (as PD-1 and PD-L1) were more sensitive to antigenic degradation over the years and estimated that histological blocks stored beyond 15 years possessed worse quality for sample study [53]. The samples from our present study are more recent, and their results could be compared with similar studies.

PD-1 expression was also associated with a lower median DOI (*p* = 0.017). Higher values of DOI were associated with higher risks of regional recurrence and metastasis in OSCC [54]. We did not find the aforementioned association between DOI and survival, and although PD-1-positive cases were associated with a lower median DOI, the multivariate model supports the independent role of PD-1 survival, so it cannot explain the lower risk of regional recurrence present in positive PD-1 cases due to a lower DOI. On the other hand, the positive expression of PD-L1 was associated with an increased risk of local recurrence (*p* = 0.074), unrelated to DOI.

Regarding the relationship of biomarkers with the histologic risk assessment score, positivity for PD-1 tended to be associated with a lower score (42% vs. 15% with risk score 0–1, *p* = 0.17), although the difference was not significant. The risk score was also not correlated with the rest of the biomarkers analyzed or survival.

Positive PD-1 and positive PD-L1 showed some tendency toward more favorable WPOI, but the differences were not significant. In the case of the WPOI, type 5 was associated with a worse prognosis in DSS (*p* = 0.075), and for OS, WPOI 4-5 yielded a negative prognosis (*p* = 0.05) when compared with the other categories. The point estimate did not change much in the multivariate models, so we cannot rule out that it has some prognostic relevance, although our study does not demonstrate this due to lack of statistical power.

The study of TILs through intratumoral CD4 and CD8 did not reveal a relationship with survival. Previous studies reported a direct relationship between the expression of CD4 and PD-L1 [23,28,36,38], which is associated with longer survival [24] and smaller tumors [38]. Our study had an average CD4 biomarker expression of 12%, and its expression was not correlated with survival or PD-L1 expression; however, it was directly but not significantly correlated with CD8 and FoxP3 (rho = 0.34).

FoxP3 plays a key role in the immunosuppressive functions of Tregs and is a marker of these cells [55]. The presence of these factors in different neoplasms is associated with a worse prognosis and with a greater risk of progression [55,56]. However, other studies linked it to a better prognosis. These contradictory findings may be related to the study of FoxP3 at the cytoplasmic vs. nuclear level [57]. In the present study, the FoxP3 biomarker was recorded at the nuclear level, and its expression was not related to OSCC prognosis.

The presence of TAM was correlated with lower OS [58]. CSF1R had a median expression of approximately 7% in the OSCC samples of the present study. We found a weak direct correlation between the expression of CSF1R and PD-L1 (Spearman’s rho *p* = 0.28). These results show some similarity with the findings of Suárez-Sánchez et al., which reflects that TAMs, identified by the expression of CD68 and CD163, were abundant in those cases with PD-L1 expression [59]. Although previous studies reported a relationship between TAMs and worse survival in other tumors [60], our research did not find such a relationship in any of the models analyzed.

We found p16 positivity in only two cases (3.1%), a prevalence that coincides with the published literature [61]. Although meta-analyses on the coexpression of PD-1 and HPV in head and neck tumors show an increase in HPV positivity [62,63], we cannot establish that such a relationship exists in OSCC due to its low HPV prevalence.

Although relevant results were found in this study regarding survival in the PD-1 and PD-L1 biomarkers, these results could be better clarified with a larger sample size. The same is true of similar studies, given the low prevalence of OSCC. The sample size decreases the statistical power of comparisons, so some relationships may not have been detected. However, this study does report an important series of cases from a single hospital with its own database, avoiding some of the limitations that arise during the collection of relevant variables and improving internal validity. Although this study has a longer follow-up time than similar studies [24,26,29,31,33,34,35,37,40,44,45], an even longer follow-up time would allow a better assessment of the results obtained. The evaluation of the samples with different biomarkers allowed us to more fully establish the TME. However, we consider that colocalizing biomarkers to specifically identify some expressed immunological cells and discern between their stromal or intratumoral location can yield important results that should be explored in future studies. To avoid leaving any relevant variable outside the multivariate model, the decision to include or not include a variable addressed not only the statistical relationship but also the biological relevance. Although we consider the region of the floor of the mouth and tongue to be very representative of the OSCC, as they are the two main locations, these results should be studied and extrapolated with caution to other oral anatomical locations, and we also consider that the results obtained require external validation through future studies.

We can confirm that the positive expression of PD-1 suggests its role as a prognostic biomarker of OSCC, as well as the positivity of PD-L1. However, these results may question current anti-PD-1 and anti-PD-L1 therapies. With the limitations of this study, the observational nature of the study and the lack of statistical power aim us to be cautious in the interpretation of this finding that contradicts previous studies; therefore, we cannot affirm its value as a predictive biomarker for this type of treatment.

Patients with PD-L1 CPS ≥ 1 and CPS ≥ 20 showed better responses to anti-PD-1 therapies [64]. This indicates that the role of positively expressed PD-L1 is related to good predictive results to treatment. However, the expression of PD-L1 is dynamic; it can change depending on the moment in which it is studied since its expression is not similar at the time of diagnosis. In a recurring or advanced disease, this heterogeneity makes it difficult to understand its prognostic role, predictions, and response to the contradictory results in different studies, as previously indicated [64]. Trying to explain our results, we considered that the presence of positive PD-1 expressed in the immune cells in the tumor could indicate that a major attempt of the immune response against the tumor from the host exists. This immune response could be related to the immunoediting cancer process, where we can find three phases, elimination, equilibrium, and tumor escape [65]. During these phases, the expression of the immune molecules could change [65]; therefore, the PD-1 expression may anticipate a future increase of PD-L1 expression before the escape phase.

The fact that the positive expression of PD-1 acts as a biomarker of good prognosis of the OSCC may suggest an approach regarding the use of anti-PD-1 therapies since sometimes the effect of a prognostic biomarker can be neutralized with the use of therapies [66]; however, a greater number of clinical trials are needed that take into account the expression of PD-1 to be able to confirm these data and to know its role as a predictive biomarker in anti-PD-1.

## 5. Conclusions

PD-1 is a protective survival factor that is maintained independently of PD-L1 expression. High values of PD-L1 expression also improve survival, while low values are associated with a worse prognosis. Higher expression of PD-1 is observed in smaller tumors, and higher expression of PD-L1 is more likely in women. At the histological level, positive PD-1 expression was associated with a lower DOI. There is no evidence of a relationship between the histological risk score and the studied biomarkers, although positive PD-1 and PD-L1 cases have a lower risk of a high WPOI score.

## Figures and Tables

**Table 1:** Descriptive statistics of clinical and histopathological variables according to sex.

Variable	N	Overall, N = 65	Sex		*p*-Value ^1^
			Men, N = 40	Women, N = 25	
Age at diagnosis	65				0.178
Mean (SD)		65 (13)	64 (13)	68 (13)	
Tobacco use	63				0.001
Never smoker		30 (48%)	11 (29%)	19 (76%)	
Former smoker		16 (25%)	13 (34%)	3 (12%)	
Current smoker		17 (27%)	14 (37%)	3 (12%)	
Alcohol use	60				<0.001
Nondrinker		41 (68%)	18 (50%)	23 (96%)	
Former drinker		7 (12%)	7 (19%)	0 (0%)	
Current drinker		12 (20%)	11 (31%)	1 (4.2%)	
Mouth Primary Location	65	23 (35%)	16 (40%)	7 (28%)	0.325
Tongue Primary Location	65	48 (74%)	26 (65%)	22 (88%)	0.040
Tumor status	63				0.162
T1		13 (21%)	8 (21%)	5 (20%)	
T2		25 (40%)	11 (29%)	14 (56%)	
T3		13 (21%)	10 (26%)	3 (12%)	
T4		12 (19%)	9 (24%)	3 (12%)	
Nodal status	60				0.902
N0		36 (60%)	21 (60%)	15 (60%)	
N1		8 (13%)	4 (11%)	4 (16%)	
N2		14 (23%)	9 (26%)	5 (20%)	
N3		2 (3.3%)	1 (2.9%)	1 (4.0%)	
Metastasis status	60				0.688
M0		53 (88%)	30 (86%)	23 (92%)	
M1		7 (12%)	5 (14%)	2 (8.0%)	
Stage	63				0.656
Stage I		8 (13%)	6 (16%)	2 (8.0%)	
Stage II		13 (21%)	7 (18%)	6 (24%)	
Stage III		28 (44%)	18 (47%)	10 (40%)	
Stage IV		14 (22%)	7 (18%)	7 (28%)	
Histological Grade	65				0.513
Grade 1: WD		22 (34%)	12 (30%)	10 (40%)	
Grade 2: MD		37 (57%)	25 (62%)	12 (48%)	
Grade 3: PD		6 (9.2%)	3 (7.5%)	3 (12%)	
Oral potentially malignant disorders	65	10 (15%)	4 (10%)	6 (24%)	0.165
Lymphoplasmacytic invasion	65				0.242
Nil		2 (3.1%)	1 (2.5%)	1 (4.0%)	
Low		33 (51%)	24 (60%)	9 (36%)	
Moderate		25 (38%)	12 (30%)	13 (52%)	
Intense		5 (7.7%)	3 (7.5%)	2 (8.0%)	
Vascular invasion	65	5 (7.7%)	4 (10%)	1 (4.0%)	0.641
Perineural invasion	65	26 (40%)	19 (48%)	7 (28%)	0.118
WPOI	65				0.275
WPOI 1		4 (6.2%)	4 (10%)	0 (0%)	
WPOI 2		30 (46%)	16 (40%)	14 (56%)	
WPOI 3		20 (31%)	12 (30%)	8 (32%)	
WPOI 4		4 (6.2%)	2 (5.0%)	2 (8.0%)	
WPOI 5		7 (11%)	6 (15%)	1 (4.0%)	
Risk Score	65				0.029
0–1		24 (37%)	13 (32%)	11 (44%)	
2–3		18 (28%)	8 (20%)	10 (40%)	
4–7		23 (35%)	19 (48%)	4 (16%)	
Depth of invasion	63				0.444
Median (IQR)		9 (3, 12)	10 (4, 12)	8 (3, 10)	
Depth of invasion	63				0.278
Less invasive		22 (35%)	13 (33%)	9 (38%)	
Moderate invasive		18 (29%)	9 (23%)	9 (38%)	
Deeply invasive		23 (37%)	17 (44%)	6 (25%)	
Local recurrence	65	19 (29%)	9 (22%)	10 (40%)	0.131
Regional recurrence	65	6 (9.2%)	4 (10%)	2 (8.0%)	>0.999
Distant recurrence	65	2 (3.1%)	1 (2.5%)	1 (4.0%)	>0.999
DFS outcome	65				0.152
Alive and without recurrence		26 (40%)	14 (35%)	12 (48%)	
Death		5 (7.7%)	4 (10%)	1 (4.0%)	
Death OSCC		11 (17%)	10 (25%)	1 (4.0%)	
Local recurrence		18 (28%)	9 (22%)	9 (36%)	
Regional or Nodal recurrence		5 (7.7%)	3 (7.5%)	2 (8.0%)	
DFS event	65	34 (52%)	22 (55%)	12 (48%)	0.583
DSS outcome	65				0.081
Alive with or without recurrence		33 (51%)	16 (40%)	17 (68%)	
Death		5 (7.7%)	4 (10%)	1 (4.0%)	
Death OSCC		27 (42%)	20 (50%)	7 (28%)	
DSS event	65	27 (42%)	20 (50%)	7 (28%)	0.080
OS Outcome	65				0.028
Alive with or without recurrence		33 (51%)	16 (40%)	17 (68%)	
Death by any cause		32 (49%)	24 (60%)	8 (32%)	
Overall Survival event	65	32 (49%)	24 (60%)	8 (32%)	0.028

^1^ Wilcoxon rank-sum test; Pearson’s Chi-squared test; Fisher’s exact test. Abbreviations: IQR: interquartile range, DFS: Disease-free survival, DSS: Disease-specific survival, MD: Moderately differentiated, OSCC: Oral squamous cell carcinoma, OS: Overall survival PD: Poorly differentiated, SD: standard deviation, WD: Well differentiated, WPOI: worst pattern of invasion.

**Table 2:** Univariate analysis of biomarkers for the different survival rates (OS, DSS, DFS).

Cox Proportional Hazard Model						
	OS: HR (CI 95%)	*p*-Value	DSS: HR (CI 95%)	*p*-Value	DFS: HR (CI 95%)	*p*-Value
PD-1 expression						
PD-1-Negative 0%	Ref.Cat.	-	Ref.Cat.	-	Ref.Cat.	-
PD-1-Positive > 0%	0.47 (0.22–1.00)	0.050	0.43 (0.19–0.98)	0.044	0.47 (0.22–0.99)	0.047
PD-L1 CPS						
[0, 1)	Ref.Cat.	-	Ref.Cat.	-	Ref.Cat.	-
[1, 20)	0.78 (0.36–1.67)	0.522	0.94 (0.43–2.01)	0.873	0.59 (0.23–1.51)	0.276
[20, 100)	0.25 (0.08–0.803)	0.019	0.59 (0.23–1.51)	0.276	0.23 (0.06–0.82)	0.024
PD-L1 CPS cutoff > 1%						
PD-L1 Negative CPS < =1%	Ref.Cat.	-	Ref.Cat.	-	Ref.Cat.	-
PD-L1 Positive CPS > 1	0.44 (0.21–0.92)	0.031	0.53 (0.24–1.17)	0.119	0.89 (0.45–1.76)	0.749
PD-L1 TPS cutoff 10%						
PD-L1 ≤ 10%	Ref.Cat.	-	Ref.Cat.	-	Ref.Cat.	-
PD-L1 > 10%	0.36 (0.14–0.94)	0.037	0.33 (0.11–0.98)	0.046	0.71 (0.32–1.57)	0.401
PD_L1 TPS cutoff 5%						
PD-L1 < 5%	Ref.Cat.	-	Ref.Cat.	-	Ref.Cat.	-
PD-L1 ≥ 5%	0.41 (0.17–0.95)	0.038	0.42 (0.16–1.05)	0.063	0.80 (0.39–1.65)	0.549
PD-L1 TPS cutoffs 5%/10%						
PD-L1 < 5%	Ref.Cat.	-	Ref.Cat.	-	Ref.Cat.	-
PD-L1 [5–10%)	1.01 (0.24–4.30)	0.992	1.26 (0.29–5.46)	0.757	0.88 (0.21–3.74)	0.857
PD-L1 [10–100%]	0.32 (0.12–0.86)	0.024	0.31 (0.10–0.91)	0.033	0.79 (0.36–1.71)	0.549
PD-L1 intensity						
Nil	Ref.Cat.	-	Ref.Cat.	-	Ref.Cat.	-
Low	0.50 (0.20–1.23)	0.134	0.53 (0.20–1.41)	0.204	0.80 (0.34–1.86)	0.603
Moderate	0.43 (0.17–1.05)	0.063	0.45 (0.17–1.20)	0.112	0.62 (0.26–1.48)	0.280
Intense	0.21 (0.03–1.66)	0.139	0.26 (0.03–2.08)	0.204	1.02 (0.29–3.57)	0.968
FOXP3						
[0.02,0.1]	Ref.Cat.	-	Ref.Cat.	-	Ref.Cat.	-
(0.1,0.15]	0.75 (0.32–1.77)	0.516	0.65 (0.25–1.71)	0.384	0.73 (0.31–1.71)	0.472
(0.15,0.3]	0.73 (0.31–1.73)	0.478	0.73 (0.29–1.84)	0.509	1.03 (0.47–2.29)	0.936
CD4						
[0.05,0.25)	Ref.Cat.	-	Ref.Cat.	-	Ref.Cat.	-
[0.25,0.35)	0.80 (0.29–2.20)	0.674	0.84 (0.27–2.58)	0.757	1.37 (0.56–3.34)	0.484
[0.35,0.50]	0.78 (0.34–1.81)	0.569	1.01 (0.42–2.45)	0.976	1.01 (0.46–2.21)	0.984
P16						
Negative	Ref.Cat.	-	Ref.Cat.	-	Ref.Cat.	-
Positive	4.45 (1.04–19.05)	0.044	2.69 (0.36–20.13)	0.337	1.29 (0.17–9.48)	0.803
CSF1R						
[0–8)%	Ref.Cat.	-	Ref.Cat.	-	Ref.Cat.	-
[8–100)%	1.17 (0.57–2.38)	0.667	1.03 (0.47–2.26)	0.944	1.00 (0.50–1.98)	0.992
CD8						
CD8 [0–10%) Mild	Ref.Cat.	-	Ref.Cat.	-	Ref.Cat.	-
CD8 [10–50)% Moderate	1.51 (0.35–6.45)	0.582	2.59 (0.34–19.53)	0.358	1.60 (0.38–6.74)	0.522
CD8 ≥ 50% Severe	1.19 (0.21–6.62)	0.849	2.44 (0.27–22.50)	0.430	1.37 (0.26–7.09)	0.711

Abbreviations: CI: Confidence Interval, DFS: Disease-free survival, DSS: Disease-specific survival, HR: Hazard Ratio, OS: Overall survival.

**Table 3:** Multivariate analysis of clinical and histopathological variables for the different survival rates (OS, DSS, DFS).

Cox Proportional Hazard Model						
	OS: HR (CI 95%)	*p*-Value	DSS: HR (CI 95%)	*p*-Value	DFS: HR (CI 95%)	*p*-Value
Gender						
Men	Ref.Cat.	-	Ref.Cat.	-	Ref.Cat.	-
Women	0.49 (0.18–1.31)	0.157	0.52 (0.18–1.47)	0.215	1.01 (0.44–2.30)	0.988
Tobacco use						
Never smoker	Ref.Cat.	-	Ref.Cat.	-	Ref.Cat.	-
Former smoker	1.77 (0.51–6.06)	0.367	2.71 (0.75–9.81)	0.128	1.61 (0.59–4.40)	0.350
Current smoker	1.12 (0.35–3.51)	0.850	1.24 (0.36–4.27)	0.738	0.64 (0.23–1.76)	0.385
Tumor status						
T1 + T2	Ref.Cat.	-	Ref.Cat.	-	Ref.Cat.	-
T3 + T4	1.10 (0.37–3.33)	0.861	0.99 (0.32–3.11)	0.990	1.30 (0.50–3.40)	0.586
Metastasis status						
M0	Ref.Cat.	-	Ref.Cat.	-	Ref.Cat.	-
M1	8.49 (1.90–37.97)	0.005	9.39 (1.85–47.60)	0.007	6.53 (1.59–26.83)	0.009
Histological Grade						
Grade 1: WD	Ref.Cat.	-	Ref.Cat.	-	Ref.Cat.	-
Grade2 + 3:MD + PD	2.96 (1.06–8.29)	0.039	5.29 (1.48–18.96)	0.010	3.46 (1.32–9.03)	0.011
Stage						
Stage I	Ref.Cat.	-	Ref.Cat.	-	Ref.Cat.	-
Stage II	1.14 (0.18–7.05)	0.887	1.98 (0.20–19.70)	0.558	0.72 (0.16–3.22)	0.667
Stage III	1.16 (0.20–6.88)	0.870	2.07 (0.21–20.26)	0.533	0.65 (0.14–2.97)	0.582
Stage IV	1.25 (0.23–6.77)	0.793	1.98 (0.22–18.06)	0.543	0.99 (0.25–4.02)	0.994
WPOI						
WPOI 1 + 2 + 3 + 4	Ref.Cat.	-	Ref.Cat.	-	Ref.Cat.	-
WPOI 5	2.47 (0.56–10.90)	0.232	3.41 (0.74–15.79)	0.117	2.14 (0.51–9.03)	0.301
PD-1						
PD-1-Negative < 0%	Ref.Cat.	-	Ref.Cat.	-	Ref.Cat.	-
PD-1-Positive > 0%	0.54 (0.20–1.48)	0.232	0.52 (0.18–1.51)	0.232	0.36 (0.14–0.93)	0.034
PD-L1 TPS cutoff 10%						
PD-L1 ≤ 10%	Ref.Cat.	-	Ref.Cat.	-	Ref.Cat.	-
PD-L1 > 10%	0.35 (0.11–1.14)	0.081	0.40 (0.12–1.35)	0.139	0.87 (0.35–2.13)	0.757

Abbreviations: CI: Confidence Interval, DFS: Disease-free survival, DSS: Disease-specific survival, HR: Hazard Ratio, MD: Moderately differentiated, OS: Overall survival PD: Poorly differentiated, SD: standard deviation, WD: Well-differentiated, WPOI: worst pattern of invasion.

## Data Availability

The data presented in this study are available on request from the corresponding author. The data are not publicly available due to privacy restrictions.

## Supplementary Materials

The following supporting information can be downloaded at: https://www.mdpi.com/article/10.3390/biomedicines10030710/s1, Figure S1: Flow chart of the selected sample, Table S1: Descriptive statistics of biomarkers according to sex, Table S2: Bivariate correlation between biomarkers Spearman’s rank-order, Table S3: Descriptive statistics of clinical and histopathological variables according to PD-1 expression and PD-L1 expression in TPS and CPS, Table S4: Univariate analysis of clinical and histopathological variables for the different survival rates (OS, DSS, DFS).
