# Supplementary material for: Prognosis Value of Immunoregulatory Molecules in Oral Cancer Microenvironment: An Immunohistochemical Study
Source: Biomedicines. 2022 Mar 19;10(3):710. doi: 10.3390/biomedicines10030710 (PMC8945047; doi:10.3390/biomedicines10030710)
Supplement: Supplementary file 1 [file biomedicines-10-00710-s001.zip › biomedicines-1611968-supplementary.pdf]

SUPPLEMENTARY INFORMATION

Supplementary Figure S1. Flow chart of the selected sample.

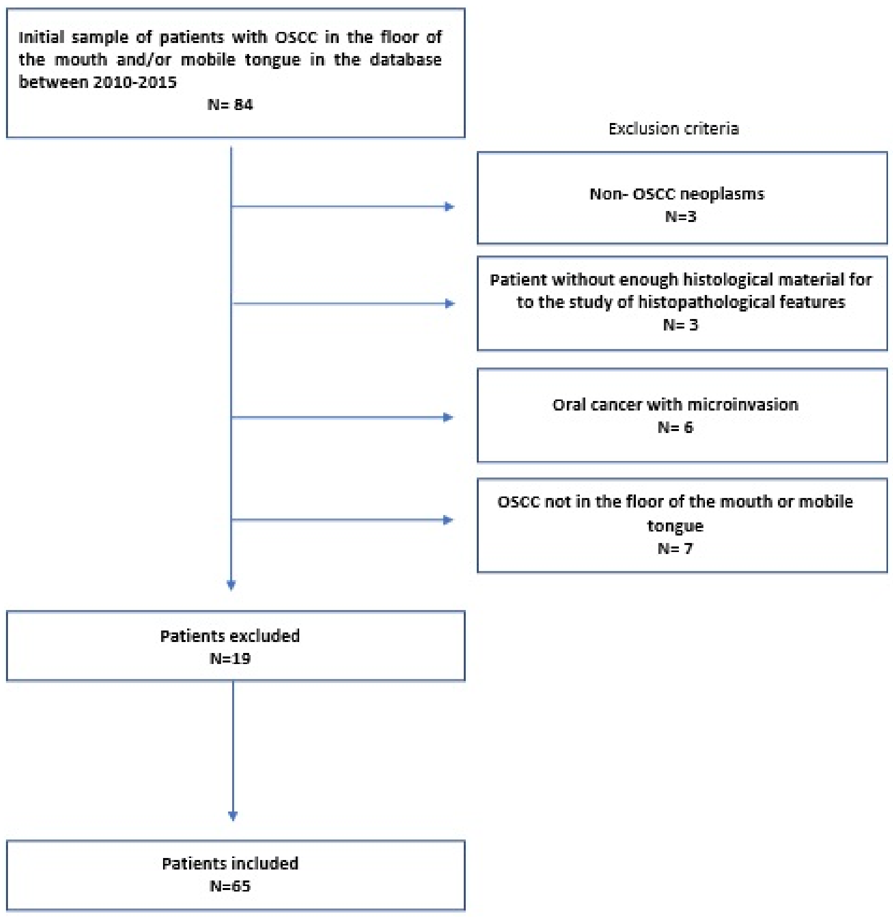

Supplementary Table S1. Descriptive statistics of biomarkers according to sex.

Variable	N	Overall, N = 65	Sex		p-value[1]
			Men, N = 40	Women, N = 25	
PD-1	65				0.838
Median (IQR)		0.01 (0.00, 0.05)	0.02 (0.00, 0.05)	0.01 (0.00, 0.03)	
PD-1	65				0.202
PD-1-Negative <0%		13 (20%)	10 (25%)	3 (12%)	
PD-1-Positive >0%		52 (80%)	30 (75%)	22 (88%)	
PD-L1 CPS	65				0.138
Median (IQR)		2 (0, 15)	1 (0, 6)	3 (1, 22)	
PD-L1 CPS	65				0.279
cps[0,1)		26 (40%)	15 (38%)	11 (44%)	
cps[1,20)		16 (25%)	8 (20%)	8 (32%)	
cps[20,100]		23 (35%)	17 (42%)	6 (24%)	
PD-L1 CPS cutoff >1	65				0.194
PD-L1 Negative CPS<=1		30 (46%)	21 (52%)	9 (36%)	
PD-L1 Positive CPS>1		35 (54%)	19 (48%)	16 (64%)	
PD-L1 intensity	65				0.250
Nil		25 (38%)	19 (48%)	6 (24%)	
Low		18 (28%)	10 (25%)	8 (32%)	
Moderate		18 (28%)	9 (22%)	9 (36%)	
Intense		4 (6.2%)	2 (5.0%)	2 (8.0%)	
PD-L1 TPS	65				0.037
Median (IQR)		0 (0, 19)	0 (0, 4)	1 (0, 40)	
PD-L1 TPS cutoffs 5%/10%	65				0.131
PD-L1 <5%		43 (66%)	30 (75%)	13 (52%)	
PD-L1 [5-10%)		4 (6.2%)	2 (5.0%)	2 (8.0%)	
PD-L1[10-100%]		18 (28%)	8 (20%)	10 (40%)	
PD-L1 TPS cutoff 5%	65				0.057
PD-L1 TPS<5%		43 (66%)	30 (75%)	13 (52%)	
PD-L1 TPS>=5%		22 (34%)	10 (25%)	12 (48%)	
PD-L1 TPS cutoff 10%	65				0.153
PD-L1 TPS <=10%		48 (74%)	32 (80%)	16 (64%)	
PD-L1 TPS >10%		17 (26%)	8 (20%)	9 (36%)	
FOXP3 (%)	65				0.073
Median (IQR)		15 (10, 20)	10 (7, 16)	15 (10, 20)	
FOXP3 cutoff 10%	65				0.033
[0-10]%		29 (45%)	22 (55%)	7 (28%)	
(10-100]%		36 (55%)	18 (45%)	18 (72%)	
CD4 (%)	65				0.223
Median (IQR)		25 (15, 35)	20 (15, 31)	30 (20, 35)	
CD4	65				0.126
[0.05,0.25)		31 (48%)	23 (57%)	8 (32%)	
[0.25,0.35)		13 (20%)	7 (18%)	6 (24%)	
[0.35,0.50]		21 (32%)	10 (25%)	11 (44%)	
P16	65				>0.999
Negative		63 (97%)	39 (98%)	24 (96%)	
Positive		2 (3.1%)	1 (2.5%)	1 (4.0%)	
CSF1R (%)	65				0.619
Median (IQR)		7 (3, 10)	7 (3, 10)	7 (3, 10)	
CSF1R	65				0.217
[0-8%)		38 (58%)	21 (52%)	17 (68%)	
[8-100]		27 (42%)	19 (48%)	8 (32%)	
CD8 (%)	65				0.644

Supplementary Table S1. Descriptive statistics of biomarkers according to sex.

Variable	N	Overall, N = 65	Sex		p-value[1]
			Men, N = 40	Women, N = 25	
Median (IQR)		30 (20, 40)	32 (20, 41)	30 (25, 35)	
CD8	65				0.099
CD8 [0-10%) Mild		5 (7.7%)	4 (10%)	1 (4.0%)	
CD8 [10-50%] Moderate		51 (78%)	28 (70%)	23 (92%)	
CD8 >=50% Severe		9 (14%)	8 (20%)	1 (4.0%)	

[1]Wilcoxon rank sum test; Pearson's Chi-squared test; Fisher's exact test

Abbreviations: IQR: interquartile range.

Supplementary Table S2. Bivariate correlation between biomarkers Spearman's rank-order correlation

Parameter 1	Parameter 2	rho (CI95%)	*p-value*
PD-1	PD-L1	0.251 (0.001-0.472)	0.737
PD-1	FOXP3	0.065 (−0.189-0.311)	1.000
PD-1	CSF1R	0.079 (−0.175-0.324)	1.000
PD-1	CD4	0.092 (−0.163-0.335)	1.000
PD-1	CD8	−0.080 (−0.325-0.174)	1.000
PD-L1	FOXP3	0.038 (−0.215-0.286)	1.000
PD-L1	CSF1R	0.283 (0.034-0.498)	0.452
PD-L1	CD4	−0.160 (−0.395-0.094)	1.000
PD-L1	CD8	0.018 (−0.234-0.268)	1.000
FOXP3	CSF1R	0.196 (−0.058-0.426)	1.000
FOXP3	CD4	0.341 (0.099-0.545)	0.114
FOXP3	CD8	0.049 (−0.204-0.296)	1.000
CSF1R	CD4	0.014 (−0.238-0.264)	1.000
CSF1R	CD8	0.016 (−0.236-0.266)	1.000
CD4	CD8	0.243 (−0.008-0.465)	0.818

Abbreviations: CI: Confidence Interval.

Supplementary Table S3. Descriptive statistics of clinical and histopathological variables according to PD-1 expression and PD-L1 expression in TPS and CPS.

Variable	N	Overall, N = 65	PD-1 Expression		p-value[1]	PD-L1 Expression TPS		p-value[1]	PD-L1 Expression CPS		p-value[1]
			Negative, <0% = 13	Positive,> >0% N = 52		TPS <5%	TPS≥5%		CPS <= 1	CPS > 1	
Sex	65				0.202			0.057			0.194
Men		40 (62%)	10 (77%)	30 (58%)		30 (70%)	10 (45%)		21 (70%)	19 (54%)	
Women		25 (38%)	3 (23%)	22 (42%)		13 (30%)	12 (55%)		9 (30%)	16 (46%)	
Age at diagnosis	65				0.254			0.598			0.171
Mean (SD)		67 (57, 73)	65 (56, 68)	68 (59, 75)		66 (56, 73)	68 (59, 76)	0.598	66 (55, 71)	69 (60, 74)	
Tobacco use	63				0.539			0.405			0.249
Never smoker		30 (48%)	4 (33%)	26 (51%)		18 (42%)	12 (60%)		12 (40%)	18 (55%)	
Former smoker		16 (25%)	4 (33%)	12 (24%)		12 (28%)	4 (20%)		7 (23%)	9 (27%)	
Current smoker		17 (27%)	4 (33%)	13 (25%)		13 (30%)	4 (20%)		11 (37%)	6 (18%)	
Alcohol use	60				0.685			0.910			>0.999
Nondrinker		41 (68%)	7 (58%)	34 (71%)		27 (66%)	14 (74%)		20 (69%)	21 (68%)	
Former drinker		7 (12%)	2 (17%)	5 (10%)		5 (12%)	2 (11%)		3 (10%)	4 (13%)	
Current drinker		12 (20%)	3 (25%)	9 (19%)		9 (22%)	3 (16%)		6 (21%)	6 (19%)	
Mouth Primary Location	65	23 (35%)	5 (38%)	18 (35%)	>0.999	18 (42%)	5 (23%)	0.127	17 (57%)	6 (17%)	<0.001
Tongue Primary Location	65	48 (74%)	11 (85%)	37 (71%)	0.486	30 (70%)	18 (82%)	0.296	18 (60%)	30 (86%)	0.019
Tumor Size status	63				0.157			0.518			0.633
T1		13 (21%)	2 (17%)	11 (22%)		10 (24%)	3 (14%)		5 (17%)	8 (24%)	
T2		25 (40%)	2 (17%)	23 (45%)		14 (33%)	11 (52%)		10 (34%)	15 (44%)	
T3		13 (21%)	4 (33%)	9 (18%)		10 (24%)	3 (14%)		7 (24%)	6 (18%)	
T4		12 (19%)	4 (33%)	8 (16%)		8 (19%)	4 (19%)		7 (24%)	5 (15%)	
Nodal status	60				0.200			0.143			0.756
N0		36 (60%)	5 (45%)	31 (63%)		27 (68%)	9 (45%)		16 (59%)	20 (61%)	
N1		8 (13%)	3 (27%)	5 (10%)		5 (12%)	3 (15%)		4 (15%)	4 (12%)	
N2		14 (23%)	2 (18%)	12 (24%)		8 (20%)	6 (30%)		7 (26%)	7 (21%)	
N3		2 (3.3%)	1 (9.1%)	1 (2.0%)		0 (0%)	2 (10%)		0 (0%)	2 (6.1%)	
Metastasis status	60				0.330			0.404			0.222
M0		53 (88%)	8 (80%)	45 (90%)		33 (85%)	20 (95%)		21 (81%)	32 (94%)	
M1		7 (12%)	2 (20%)	5 (10%)		6 (15%)	1 (4.8%)		5 (19%)	2 (5.9%)	
Stage	63				0.574			0.612			0.680
Stage I		8 (13%)	1 (8.3%)	7 (14%)		7 (17%)	1 (4.8%)		4 (14%)	4 (12%)	
Stage II		13 (21%)	3 (25%)	10 (20%)		9 (21%)	4 (19%)		5 (17%)	8 (24%)	
Stage III		28 (44%)	7 (58%)	21 (41%)		17 (40%)	11 (52%)		15 (52%)	13 (38%)	
Stage IV		14 (22%)	1 (8.3%)	13 (25%)		9 (21%)	5 (24%)		5 (17%)	9 (26%)	
Histological Grade	65				0.478			0.239			0.456
Grade 1: WD		22 (34%)	4 (31%)	18 (35%)		17 (40%)	5 (23%)		11 (37%)	11 (31%)	
Grade 2: MD		37 (57%)	9 (69%)	28 (54%)		21 (49%)	16 (73%)		15 (50%)	22 (63%)	
Grade 3: PD		6 (9.2%)	0 (0%)	6 (12%)		5 (12%)	1 (4.5%)		4 (13%)	2 (5.7%)	
Oral potentially malignant disorders	65	10 (15%)	0 (0%)	10 (19%)	0.191	7 (16%)	3 (14%)	>0.999	6 (20%)	4 (11%)	0.493
Lymphoplasmacytic invasion	65				0.030			0.647			0.731
Nil		2 (3.1%)	0 (0%)	2 (3.8%)		2 (4.7%)	0 (0%)		1 (3.3%)	1 (2.9%)	
Low		33 (51%)	11 (85%)	22 (42%)		20 (47%)	13 (59%)		15 (50%)	18 (51%)	
Moderate		25 (38%)	1 (7.7%)	24 (46%)		18 (42%)	7 (32%)		13 (43%)	12 (34%)	
Intense		5 (7.7%)	1 (7.7%)	4 (7.7%)		3 (7.0%)	2 (9.1%)		1 (3.3%)	4 (11%)	
Vascular invasion	65	5 (7.7%)	1 (7.7%)	4 (7.7%)	>0.999	3 (7.0%)	2 (9.1%)	>0.999	3 (10%)	2 (5.7%)	0.655
Perineural invasion	65	26 (40%)	7 (54%)	19 (37%)	0.255	19 (44%)	7 (32%)	0.335	14 (47%)	12 (34%)	0.310
WPOI	65				0.313						0.016
WPOI 1		4 (6.2%)	1 (7.7%)	3 (5.8%)		4 (9.3%)	0 (0%)	0.102	4 (13%)	0 (0%)	
WPOI 2		30 (46%)	4 (31%)	26 (50%)		17 (40%)	13 (59%)		10 (33%)	20 (57%)	
WPOI 3		20 (31%)	7 (54%)	13 (25%)		13 (30%)	7 (32%)		8 (27%)	12 (34%)	
WPOI 4		4 (6.2%)	0 (0%)	4 (7.7%)		2 (4.7%)	2 (9.1%)		2 (6.7%)	2 (5.7%)	
WPOI 5		7 (11%)	1 (7.7%)	6 (12%)		7 (16%)	0 (0%)		6 (20%)	1 (2.9%)	
Risk Score	65				0.165			0.229			0.043
0-1		24 (37%)	2 (15%)	22 (42%)		17 (40%)	7 (32%)		12 (40%)	12 (34%)	
2-3		18 (28%)	4 (31%)	14 (27%)		9 (21%)	9 (41%)		4 (13%)	14 (40%)	
4-7		23 (35%)	7 (54%)	16 (31%)		17 (40%)	6 (27%)		14 (47%)	9 (26%)	
Depth of invasion	63				0.017			0.170			0.978

Median (IQR)		9 (3, 12)	12 (7, 16)	8 (3, 11)		7 (3, 11)	10 (8, 12)		7 (3, 12)	9 (4, 12)	
Local recurrence	65	65	19 (29%)	3 (23%)	0.740	10 (23%)	9 (41%)	0.139	5 (17%)	14 (40%)	0.039
Regional recurrence	65	65	6 (9.2%)	3 (23%)	0.089	5 (12%)	1 (4.5%)	0.655	3 (10%)	3 (8.6%)	>0.999
Distant recurrence	65	65	2 (3.1%)	1 (7.7%)	0.362	2 (4.7%)	0 (0%)	0.545	1 (3.3%)	1 (2.9%)	>0.999
DFS outcome	65				0.039						
Alive and without recurrence		26 (40%)	2 (15%)	24 (46%)		16 (37%)	10 (45%)		11 (37%)	15 (43%)	
Death		5 (7.7%)	1 (7.7%)	4 (7.7%)		4 (9.3%)	1 (4.5%)		4 (13%)	1 (2.9%)	
Death OSCC		11 (17%)	4 (31%)	7 (13%)		9 (21%)	2 (9.1%)		7 (23%)	4 (11%)	
Local recurrence		18 (28%)	3 (23%)	15 (29%)		10 (23%)	8 (36%)		5 (17%)	13 (37%)	
Regional or Nodal recurrence		5 (7.7%)	3 (23%)	2 (3.8%)		4 (9.3%)	1 (4.5%)		3 (10%)	2 (5.7%)	
DFS event	65	34 (52%)	10 (77%)	24 (46%)	0.047	23 (53%)	11 (50%)	0.790	15 (50%)	19 (54%)	0.730
DSS outcome	65				0.009			0.404			0.148
Alive with or without recurrence		33 (51%)	2 (15%)	31 (60%)		19 (44%)	14 (64%)		12 (40%)	21 (60%)	
Death		5 (7.7%)	1 (7.7%)	4 (7.7%)		4 (9.3%)	1 (4.5%)		4 (13%)	1 (2.9%)	
Death OSCC		27 (42%)	10 (77%)	17 (33%)		20 (47%)	7 (32%)		14 (47%)	13 (37%)	
DSS event	65	34 (52%)	10 (77%)	24 (46%)	0.004	20 (47%)	7 (32%)	0.255	14 (47%)	13 (37%)	
OS Outcome	65				0.004			0.138			0.108
Alive with or without recurrence		33 (51%)	2 (15%)	31 (60%)		19 (44%)	14 (64%)		12 (40%)	21 (60%)	
Death by any cause		32 (49%)	11 (85%)	21 (40%)		24 (56%)	8 (36%)		18 (60%)	14 (40%)	
**Overall Survival event**	65	32 (49%)	11 (85%)	21 (40%)	0.004	24 (56%)	8 (36%)	0.138	18 (60%)	14 (40%)	0.108

1 Wilcoxon rank sum test; Pearson's Chi-squared test; Fisher's exact test

Abbreviations: DFS: Disease-free survival, DSS: Disease-specific survival, MD: Moderately differentiated, OSCC: Oral squamous cell carcinoma, OS: Overall survival PD: Poorly differentiated, SD: standard deviation, WD: Well differentiated, WPOI: worst pattern of invasion.

Supplementary Table S4. Univariate analysis of clinical and histopathological variables for the different survival rates (OS, DSS, DFS)

	Cox proportional Hazard Model					
	OS: HR(CI95%)	p-value	DSS: HR(CI95%)	p-value	DFS: HR(CI95%)	p-value
Age at diagnosis						
	Ref.Cat.	-	Ref.Cat.	-	Ref.Cat.	-
NA	1.02 (0.99-1.05)	0.107	1.01 (0.98-1.04)	0.373	1.02 (0.99-1.04)	0.177
Gender						
Men	Ref.Cat.	-	Ref.Cat.	-	Ref.Cat.	-
Women	0.35 (0.15-0.82)	0.015	0.36 (0.14-0.89)	0.028	0.80 (0.40-1.63)	0.549
Tobacco use						
Never smoker	Ref.Cat.	-	Ref.Cat.	-	Ref.Cat.	-
Former smoker	1.50 (0.59-3.84)	0.395	1.96 (0.73-5.31)	0.184	1.40 (0.62-3.19)	0.418
Current smoker	2.11 (0.90-4.90)	0.084	2.47 (0.97-6.27)	0.056	1.14 (0.50-2.59)	0.757
Alcohol use						
Nondrinker	Ref.Cat.	-	Ref.Cat.	-	Ref.Cat.	-
Former drinker	1.73 (0.64-4.68)	0.280	2.07 (0.75-5.73)	0.162	1.78 (0.72-4.44)	0.211
Current drinker	1.28 (0.51-3.25)	0.596	1.25 (0.45-3.45)	0.667	1.13 (0.46-2.83)	0.787
Tumor location						
Tongue	Ref.Cat.	-	Ref.Cat.	-	Ref.Cat.	-
Base of the Mouth	0.87 (0.36-2.05)	0.741	0.92 (0.36-2.35)	0.857	0.67 (0.29-1.57)	0.358
Tongue and Base of the Mouth	1.16 (0.34-3.93)	0.810	1.39 (0.40-4.78)	0.603	1.61 (0.55-4.68)	0.384
Tumor status						
T1	Ref.Cat.	-	Ref.Cat.	-	Ref.Cat.	-
T2	1.46 (0.47-4.54)	0.516	1.43 (0.38-5.31)	0.596	1.06 (0.40-2.83)	0.912
T3	2.16 (0.63-7.41)	0.222	2.43 (0.60-9.76)	0.211	1.04 (0.34-3.23)	0.944
T4	2.29 (0.69-7.65)	0.177	3.00 (0.79-11.38)	0.107	2.21 (0.80-6.10)	0.124
Nodal Status						
N0	Ref.Cat.	-	Ref.Cat.	-	Ref.Cat.	-
N1	1.21 (0.40-3.67)	0.741	1.07 (0.30-3.84)	0.912	1.57 (0.57-4.31)	0.384
N2/N3	1.04 (0.45-2.40)	0.920	1.12 (0.46-2.76)	0.795	0.88 (0.40-1.98)	0.764
Metastasis status						
M0	Ref.Cat.	-	Ref.Cat.	-	Ref.Cat.	-
M1	5.84 (2.18-15.64)	0.000	6.00 (2.02-17.81)	0.001	3.11 (1.15-8.44)	0.026
Histological Grade						
Grade 1: WD	Ref.Cat.	-	Ref.Cat.	-	Ref.Cat.	-
Grade 2: MD	1.26 (0.54-2.95)	0.596	1.77 (0.64-4.92)	0.276	1.63 (0.72-3.71)	0.242
Grade 3: PD	5.07 (1.73-14.84)	0.003	8.09 (2.43-26.97)	0.001	5.15 (1.75-15.15)	0.003
Stage						
Stage I	Ref.Cat.	-	Ref.Cat.	-	Ref.Cat.	-
Stage II	1.43 (0.28-7.39)	0.674	2.75 (0.32-23.63)	0.358	1.41 (0.36-5.48)	0.617
Stage III	2.24 (0.52-9.74)	0.280	3.88 (0.51-29.46)	0.190	1.56 (0.46-5.36)	0.478
Stage IV	1.71 (0.35-8.29)	0.503	2.39 (0.28-20.48)	0.430	1.32 (0.34-5.12)	0.689
Oral potentially malignant disorders						
No	Ref.Cat.	-	Ref.Cat.	-	Ref.Cat.	-
Yes	0.81 (0.28-2.32)	0.697	0.46 (0.11-1.94)	0.289	0.74 (0.26-2.10)	0.569
Perineural invasion						
No	Ref.Cat.	-	Ref.Cat.	-	Ref.Cat.	-
Yes	1.21 (0.58-2.51)	0.610	1.25 (0.56-2.79)	0.575	1.04 (0.52-2.08)	0.912
Vascular invasion						
No	Ref.Cat.	-	Ref.Cat.	-	Ref.Cat.	-
Yes	0.68 (0.16-2.95)	0.610	0.81 (0.18-3.59)	0.787	0.59 (0.14-2.53)	0.478
Lymphoplasmacytic invasion						
Nil/Low	Ref.Cat.	-	Ref.Cat.	-	Ref.Cat.	-
Moderate	0.86 (0.40-1.89)	0.711	0.67 (0.28-1.64)	0.384	0.90 (0.43-1.88)	0.779
Intense	1.04 (0.24-4.61)	0.952	1.16 (0.26-5.19)	0.841	0.73 (0.17-3.15)	0.674
Margins affected						
No	Ref.Cat.	-	Ref.Cat.	-	Ref.Cat.	-
Yes	0.50 (0.07-3.71)	0.503	0.61 (0.08-4.53)	0.631	0.44 (0.06-3.22)	0.418
Risk Score						
0-1	Ref.Cat.	-	Ref.Cat.	-	Ref.Cat.	-
2-3	0.92 (0.36-2.37)	0.865	1.02 (0.37-2.86)	0.968	1.02 (0.43-2.41)	0.968
4-7	1.26 (0.54-2.94)	0.582	1.35 (0.53-3.41)	0.529	1.17 (0.52-2.63)	0.704
WPOI 1+2+3 VS 4+5						
WPOI 1+2+3	Ref.Cat.	-	Ref.Cat.	-	Ref.Cat.	-
WPOI 4+5	2.34 (1.00-5.47)	0.050	1.92 (0.72-5.12)	0.194	1.76 (0.72-4.28)	0.211
Depth of Invasion						
Less invasive	Ref.Cat.	-	Ref.Cat.	-	Ref.Cat.	-
Moderate invasive	0.93 (0.39-2.23)	0.873	0.89 (0.34-2.36)	0.818	1.07 (0.48-2.42)	0.865

Deeply invasive	0.64 (0.26-1.57)	0.332	0.69 (0.26-1.81)	0.453	0.51 (0.21-1.20)	0.124
